# Metabolomics analysis reveals the association between lipid abnormalities and oxidative stress, inflammation, fibrosis, and Nrf2 dysfunction in aristolochic acid-induced nephropathy

**DOI:** 10.1038/srep12936

**Published:** 2015-08-07

**Authors:** Ying-Yong Zhao, Hui-Ling Wang, Xian-Long Cheng, Feng Wei, Xu Bai, Rui-Chao Lin, Nosratola D. Vaziri

**Affiliations:** 1 Key Laboratory of Resource Biology and Biotechnology in Western China, Ministry of Education, the College of Life Sciences, Northwest University, No. 229 Taibai North Road, Xi’an, Shaanxi 710069, China; 2 Division of Nephrology and Hypertension, School of Medicine, University of California, Irvine, MedSci 1, C352, UCI Campus, Irvine, California, 92897, USA; 3 School of Chinese Materia Medica, Beijing University of Chinese Medicine, No. 11 North Third Ring Road, Beijing 100029, China; 4 National Institutes for Food and Drug Control, State Food and Drug Administration, No. 2 Tiantan Xili, Beijing, 100050, China; 5 Solution Centre, Waters Technologies (Shanghai) Ltd., No. 1000 Jinhai Road, Shanghai 201203, China; 6 Department of nephrology, Shanghai Jimin Hospital, No. 338 Huaihai West Road, Shanghai 200052, China

## Abstract

Alternative medicines are commonly used for the disease prevention and treatment worldwide. Aristolochic acid (AAI) nephropathy (AAN) is a common and rapidly progressive interstitial nephropathy caused by ingestion of Aristolochia herbal medications. Available data on pathophysiology and molecular mechanisms of AAN are limited and were explored here. SD rats were randomized to AAN and control groups. AAN group was treated with AAI by oral gavage for 12 weeks and observed for additional 12 weeks. Kidneys were processed for histological evaluation, Western blotting, and metabolomics analyses using UPLC-QTOF/HDMS. The concentrations of two phosphatidylcholines, two diglycerides and two acyl-carnitines were significantly altered in AAI treated rats at week 4 when renal function and histology were unchanged. Data obtained on weeks 8 to 24 revealed progressive tubulointerstitial fibrosis, inflammation, renal dysfunction, activation of NF-κB, TGF-β, and oxidative pathways, impaired Nrf2 system, and profound changes in lipid metabolites including numerous PC, lysoPC, PE, lysoPE, ceramides and triglycerides. In conclusion, exposure to AAI results in dynamic changes in kidney tissue fatty acid, phospholipid, and glycerolipid metabolisms prior to and after the onset of detectable changes in renal function or histology. These findings point to participation of altered tissue lipid metabolism in the pathogenesis of AAN.

Natural and alternative medicines are commonly used for the treatment or prevention of various diseases worldwide. However, the available data regarding their mechanisms of action and potential toxicity are limited and urgently needed. Nephrotoxicity is a serious side effect of several commonly used natural products and includes electrolyte abnormalities, proteinuria, acute kidney injury, chronic kidney disease (CKD), and death. Mechanisms of natural medicine-associated kidney injury include direct nephrotoxicity, which may be augmented by predisposing conditions such as dehydration; contamination, adulteration, inappropriate use, improper preparation, or interactions with other medications. Therefore, the underlying mechanism of renal injury and dysfunction associated with these products remains incompletely understood.

The pre-clinical drug safety evaluations for detection of nephrotoxicity rely primarily on measurements of routine serum chemical parameters including creatinine and urea concentrations, urinalysis, and renal histopathological analysis. Aristolochic acid nephropathy (AAN) is one of the most common iatrogenic kidney diseases which is caused by intake of Aristolochia herbal medications^1^. AAN is a rapidly progressive renal interstitial nephropathy that can lead to end-stage renal disease. Numerous cases of AAN have occurred in Belgium, France, Spain, UK, China and Japan from intake of slimming pills containing Aristolochia fangchi. Close to 50% of these cases required renal replacement therapy^1^. Previous toxicological studies of AAN had focused mainly on formation of metabolites and DNA adducts^1^ and animal studies had mainly focused on aristolochic acids (AA)-induced acute tubular necrosis or acute nephrotoxicity^1^. Only a few experiments demonstrated chronic AAN using AA extracts in rats or rabbits^2^,^3^,^4^. However in clinical setting AAN is caused by long-term intake of AA-containing medications. Knowledge of the pathophysiological mechanisms and complex molecular processes causing AAN remains limited to the information drawn by use of conventional investigative tools and techniques.

Use of the novel metabolomics analysis for global metabolic profiling has attracted increasing interest in the field of toxicology since it allows simultaneous, rapid and reproducible determination of the levels of endogenous metabolites that directly reflect the biological events in the test samples. The role of metabolomics in the study of nephrotoxicity has been described in a recent review^5^. Aristolochic acid I (AAI) and aristolochic acid II (AAII) are the main chemical components of Aristolochia. Both AAI and AAII are potentially carcinogenic, while only AAI is nephrotoxic^6^. In the present study, we used a standard method to induce chronic AAN in Sprague-Dawley rats in order to explore the mechanism of the associated tubulo-interstitial nephropathy. To this end we assessed kidney function, kidney histopathology, inflammatory, oxidative, anti-oxidant, and fibrotic pathways. In addition kidney tissue metabolic profile was assessed using ultra performance liquid chromatography-quadrupole time-of-flight high-definition mass spectrometry (UPLC-Q-TOF/HDMS)-based metabolomics to identify tissue biomarkers of early, intermediary and advanced stages of kidney disease in this model.

## Results

### General data

Data are summarized in Table 1. Body weight was significantly reduced and urine volume and urine pH were significantly increased in the AAN rats compared with the control rats at weeks 4 to 24. The urine specific gravity, osmolality and creatinine levels steadily declined and urine protein and sodium increased in the AAN rats during weeks 8 to 24. However, serum sodium remained unchanged in the AAN rats throughout the study period. Compared with the control rats, the AAN rats showed significant increase in plasma MDA as well as renal tissue and mitochondrial TBARS within 8 to 24 weeks. In addition, the AAN rats showed a significant increase in GSSG and GSSG/GSH ratio and a significant reduction of GSH within 8 to 24 weeks.

### Histological findings

**Figure 1:**
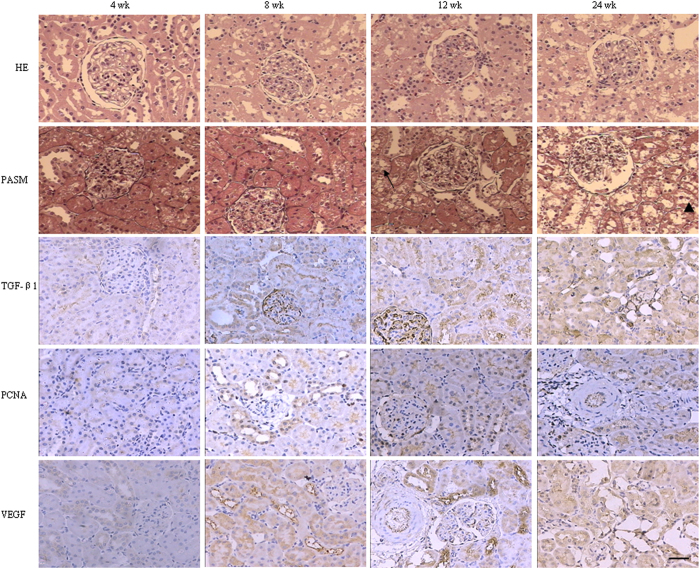
Kidney histological features of rat with oral administration. Rats were sacrificed at the indicated times after AAI exposure. Paraffin-embedded sections (5mm) were stained with haematoxylin & eosin (HE), or with periodic acid-silver metheramine (PASM). Kidney tissues were stained with HE or PASM. No morphological changes were noted for control and 4 weeks rats. Typical lesions, consisting of granular degeneration (arrowheads) and vacuolar degeneration (arrows), were observed, accompanied by upregulated expression of TGF-β1, PCAN and VEGF in AAN group within 8 to 24 weeks. Bar = 30 μm.

Representative photomicrographs of hematoxylin & Eosins (H&E) and periodic acid-Schiff (PAS) stained kidney tissues as well as immunohistological sections for transforming growth factor β1 (TGF**-**β1), proliferating cell nuclear antigen (PCNA), and vascular endothelial growth factor (VEGF) are shown in Fig. 1. No significant abnormalities were observed in the kidney tissues obtained during the study periods in control rats. Likewise no significant abnormalities were found in the kidneys of the AAN rats at week 4. In contrast, kidney tissues from the AAN rats exhibited progressive tubulo-interstitial lesions from 8 to 24 weeks. At week 8, HE and PAS staining of AAN rats’ kidneys showed tubular cell swelling, vacuolization, and slight granular degeneration, and modest monocyte and lymphocyte infiltration. At week 12, H&E and PAS staining showed extensive loss of tubular brush border, multiple foci of tubular necrosis and atrophy, inflammatory cell infiltration and interstitial fibrosis. At week 24, AAN rat showed further deterioration of tubular atrophy and renal interstitial fibrosis and accumulation of myofibroblasts. Immunohistochemical staining showed a significant increase in TGF-β1 expression in the tubular epithelial cells and glomeruli from 8 to 24 weeks. Likewise compared with the control group, PCNA expression in the renal tubules was significantly increased from 8 to 24 weeks. In addition, interstitial fibroblasts, interstitial inflammatory cells and glomeruli showed light expression of PCNA protein. Compared with the control group, the expression of VEGF in the renal tubule was significantly increased from 8 to 24 weeks.

### Oxidative, inflammatory, Nrf2, and fibrotic pathways

**Figure 2:**
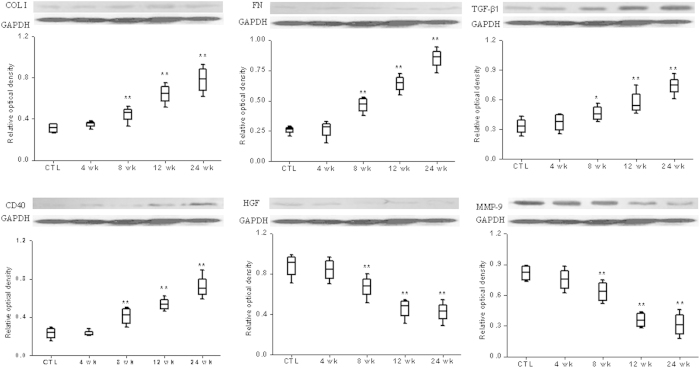
Expression of pro-fibrotic proteins in kidney from control and model rats. Representative western blots of fibrotic protein including TGF**-**β1, FN, HGF, CD40, VEGF and MMP-9 in the control and model rats in the 4, 8, 12 and 24 weeks (n = 8). GAPDH served as the loading control. *P < 0.05, **P < 0.01.

Western blotting of oxidative, inflammatory and Nrf2 are shown in Fig. S1 and S2. Compared with the control rats, the AAN rats exhibited significant increase in protein abundance of the NAD(P)H oxidase subunits NOX4, p47^phox^, p22^phox^ and 3-nitrotyrosine within 8 to 24 weeks. Similarly, COX-2, iNOS, MCP-1 and LOX-1 abundance was significantly increased in the AAN rats at 8 to 24 weeks. This was associated with a significant increase in p-IκB and nuclear translocation of p65 subunits of NF-κB, the master regulator of many pro-inflammatory and pro-fibrotic molecules. Compared with the control rat, the AAN rat exhibited a significant decrease in nuclear Nrf2 abundance and a significant increase in Keap1 abundance in cytoplasmic protein extracts. This was associated with significant decrease in HO-1, NQO1, GCLC, GCLM, Cu,Zn-SOD, Mn-SOD and catalase. Activation of inflammatory and oxidative pathways in AAN rat was accompanied by significant up-regulation of collagen I, TGF**-**β1, fibronectin and CD40 as well as significant down-regulation of hepatocyte growth factor (HGF) and MMP-9 (Fig. 2). Taken together, these findings point to activation of the pro-inflammatory, pro-oxidant, and fibrotic pathways and down-regulation of Nrf2-mediated antioxidant and phase 2 detoxifying enzymes and related proteins.

### Metabolite abnormalities

**Figure 3:**
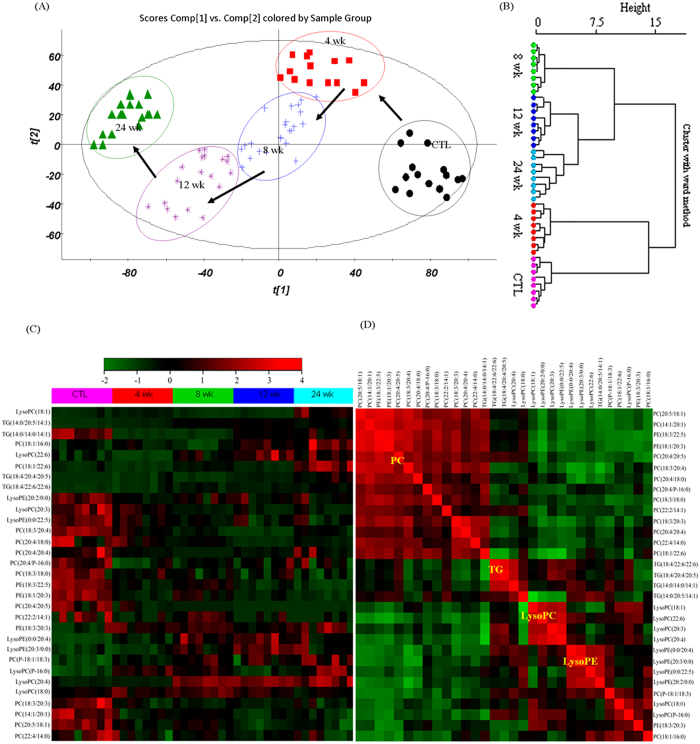
Multivariate statistical analysis of AAN group and control group. (**A**) Visualization of alterations in the metabolic trajectories in the AAN rats. Principal component analysis score plot of lipidomics profiles for the different AAN groups at weeks 0, 4, 8, 12 and 24, utilizing only the ion peaks and two classes (AAN group and control group) to build the model. Two latent variables were used. (**B**) Dendrograms of hierarchical clustering of different groups at weeks 0, 4, 8, 12 and 24 using significant metabolites by one-way ANOVA. (**C**) Hierarchical clustering heat map of the 33 differential metabolites, with the degree of change in the different AAN groups compared with the control group marked with colors including up-regulation (red) and down-regulation (green). Identified lipids were represented in the horizontal axis, and individual samples in the vertical axis. Data were log-transformed, obtained after clustering of identified lipids and individual samples, analyzed by the Ward Agglomeration Algorithm, and filtered for control group vs AAN group comparison. (**D**) Relationships of correlation matrix between clusters of 33 individual lipid species using Pearson’s linear correlation analysis. Pearson’s correlations were calculated between 33 individual lipid species marked on the plot. The color scale illustrates the magnitude and direction of correlation between individual lipid molecular species. Red and green indicated positive and negative correlations, respectively. Six clusters were identified representing the different groups of metabolites.

Metabolic profiling of kidney tissues was acquired by UPLC-MS in positive ion mode and OPLS-DA was employed for determination of kidney tissue metabolites. AAN and control groups could be separated completely in the OPLS-DA score plots (supplementary Fig. S3) indicating that kidney metabolism was significantly changed in AAN rats. PCA was performed for the identification of metabolites in various AAN groups using the MS data. The PCA score plot showed the metabolic trajectory of AAN rat at weeks 4, 8, 12 and 24 and revealed a clear separation at different time points (Fig. 3A). This demonstrated that AAI causes different metabolic perturbations paralleling different pathobiological changes in the course of AAN. Identified metabolites were selected by VIP value of S-plots (supplementary Fig. S3). Potential metabolites were identified according to the previously reported method^7^,^8^. The identified biomarkers are shown in supplementary Table S1. Heat maps showed significant differences and changes in identified metabolites in the kidney tissues from AAN and control groups at weeks 4, 8, 12 and 24 (supplementary Fig. S4). These changes were consistent with OPLS-DA loading plots of AAN and control groups (supplementary Fig. S5).

### Metabolite abnormalities in early stage of AAI nephrotoxicity

**Figure 4:**
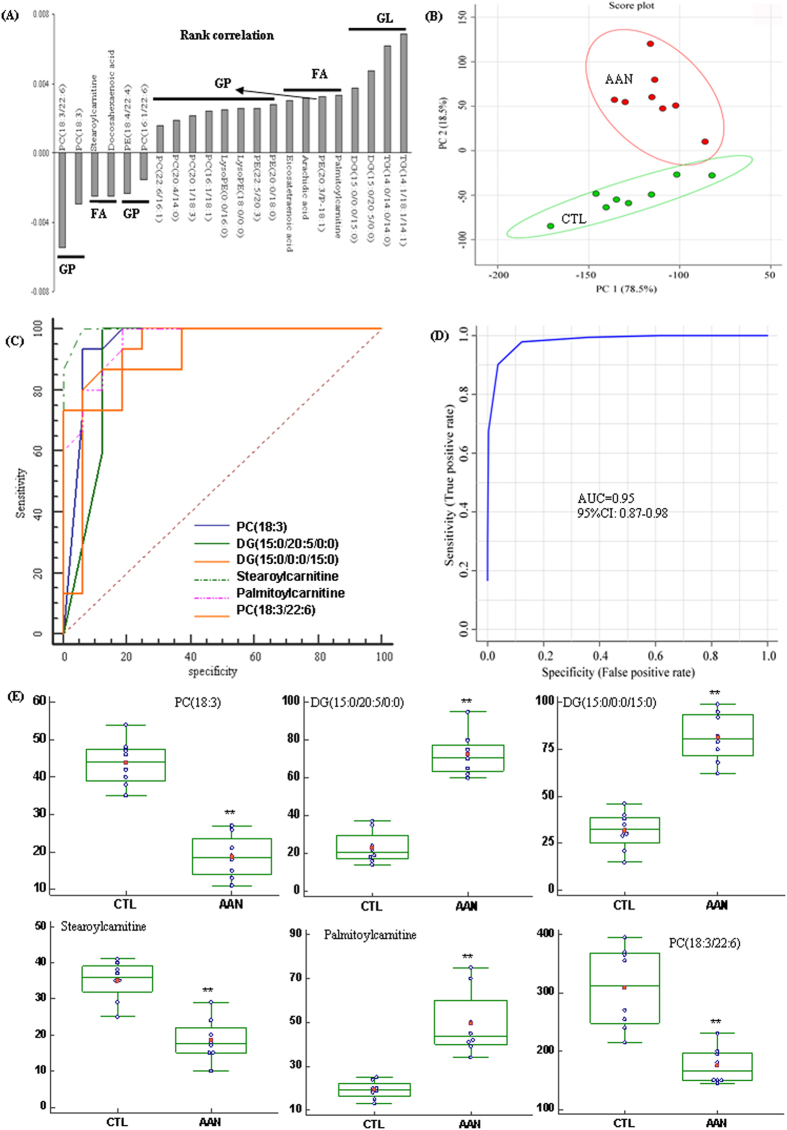
Analyses of OPLS-DA loading plot and PCA of 22 identified lipids as well as diagnostic performance and relative intensity of 6 early kidney injury biomarkers in the 4th week. (**A**) OPLS-DA loading plot of 22 differential lipids only appearing in the 4th week. Upper values with a positive correlation showed a relatively higher kidney tissue levels present in the AAN rats, whereas low value indicated a relatively lower kidney tissue levels present in the AAN rats. (**B**) PCA of 22 differential lipids. The AAN and control could be separated completely based on these 22 identified lipids. These identified lipids showed an early 4th week nephrotoxic drug-induced response in kidney tissue prior to observable histological kidney damage and conventional clinical chemistry indications of nephrotoxicity. PLS-DA-based ROC curves for the evaluation of AAN with the individual metabolites (**C**) and the combination of the six metabolites (**D**) with high sensitivity and specificity from 22 identified lipids. They could serve as suitable biomarkers for very early nephrotoxicity effects. (**E**) Combined box-and-whisker and dot plot of normalized intensity of six metabolites (PC(18:3), PC(18:3/22:6), DG(15:0/20:5/0:0), DG(15:0/0:0/15:0), stearoylcarnitine and palmitoylcarnitine) in kidney tissue of control and AAN groups. The statistical significance of differences between the two groups was marked.

No significant abnormalities including conventional clinical chemistry and histological kidney damage were observed in the kidney tissue in the AAN rats at week 4. However 22 metabolites showed significant changes at week 4 and as such could be indicative of early AAI nephrotoxicity (Fig. 4A). An analysis of the OPLS-DA loading plot conducted to identify these metabolites revealed significant increase in two TG, two DG, three fatty acids, three PE, two lysoPE and four PC as well as significant decrease in three PC, two fatty acids and one PE in the AAN rats (Fig. 4A).

Next we used two additional approaches to further explore the impact of these selected metabolites. First, PCA was performed to separate the AAN from control rats. Fig. 4B shows the PCA of 22 different metabolites from AAN rats which could be separated completely from control rats. Next, we calculated the PLS-DA-based receiver-operating characteristic ROC curves for the detection of early kidney injury with the individual metabolites. The area under the curve (AUC), 95% confidence interval (95%CI), sensitivities and specificities are summarized in Table 2. Ten metabolites including PC(18:3), DG(15:0/20:5/0:0), DG(15:0/0:0/15:0), stearoylcarnitine, palmitoylcarnitine, TG(14:1/18:1/14:1), TG(14:0/14:0/14:0), PC(18:3/22:6), PE(18:4/22:4) and PC(16:1/18:1) were identified as the top-ranked candidates, with an AUC value of more than 0.80. Although the AUC value for TG(14:1/18:1/14:1) and PC(16:1/18:1) were high, they were excluded from the subsequent validation process because their diagnostic sensitivities were low. Similarly, although TG(14:0/14:0/14:0), PE(18:4/22:4) and PC(16:1/18:1) exhibited a high diagnostic performance in the current data set, their diagnostic specificity were found to be low, and thus were not considered to be suitable tissue metabolites for predicting nephrotoxicity. PC(18:3), DG(15:0/20:5/0:0), DG(15:0/0:0/15:0), stearoylcarnitine, palmitoylcarnitine and PC(18:3/22:6) were the suitable metabolites with high sensitivity and specificity for predicting nephrotoxicity. PLS-DA-based ROC curves for the evaluation of AAN with the individual metabolites and the combination of the six metabolites are shown in Fig. 4C,D. Combined box-and-whisker and dot plot of normalized intensity of six metabolites are shown in Fig. 4E. The changes of these metabolites were most significant at week 4, and less pronounced later in the course of the disease. Therefore, the study identified these six metabolites as markers of early nephrotoxicity prior to appearance conventional biochemical or histological abnormalities.

### Metabolites associated with moderate nephrotoxicity

**Figure 5:**
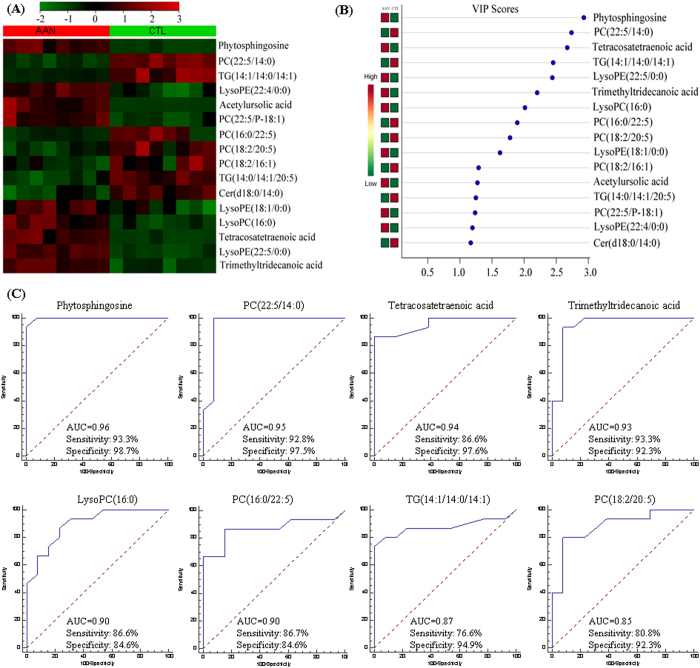
Hierarchical cluster analysis and ROC curves of 16 identified metabolites from AAN and control group in the 8th week. (**A**) Clustered heat map of only 16 (only appeared at 8 week or after 8 week) out of 69 identified metabolites between 8th week AAN and control groups after observable histological kidney damage and conventional clinical chemistry indications of nephrotoxicity. Heatmap displays of the most significantly changes from 16 metabolites. The colour of each section is proportional to the significance of alternation of metabolites in kidney tissue. Green indicates reduction, red indicates increase compared to control group. Columns represent individual experiments and rows represent each identified metabolites from AAN and control groups in the 8th week. (**B**) VIP scores with expression heat map from PLS-DA analysis. PLS-DA analysis was constructed with signature metabolites from AAN and control rats. Red and green indicated increased and decreased levels, respectively. (**C**) PLS-DA based ROC curves of 8 out of 16 identified metabolites for evaluation of AAN with the individual biomarkers. The associated AUC, sensitivity and specificity values were indicated.

Hierarchical cluster analysis and the resulting heat map revealed that among the 69 metabolites which were significantly altered at week 8 and beyond. Sixteen of these metabolites were markedly altered in the AAN group at week 8 only (Fig. 5A). The cluster diagram revealed that two groups could be separated completely by these sixteen sleeted metabolites with VIP scores of more than 1.0 (Fig. 5B). To assess the predictive performance of the selected metabolites in the AAN, we calculated the AUC value. Among the above 16 metabolites eight were identified as the top-ranked candidates, with an AUC value of more than 0.85 (Fig. 5C) including phytosphingosine, PC(22:5/14:0), tetracosatetraenoic acid, trimethyltridecanoic acid, lysoPC(16:0), PC(16:0/22:5), TG(14:1/14:0/14:1) and PC(18:2/20:5). As a result, these eight metabolites could potentially be considered as predictive metabolites corresponding with the histological evidence of kidney damage, conventional clinical chemistry and activation of inflammatory, oxidant and fibrotic pathways.

### Changes of Metabolites in advanced AAN

**Figure 6:**
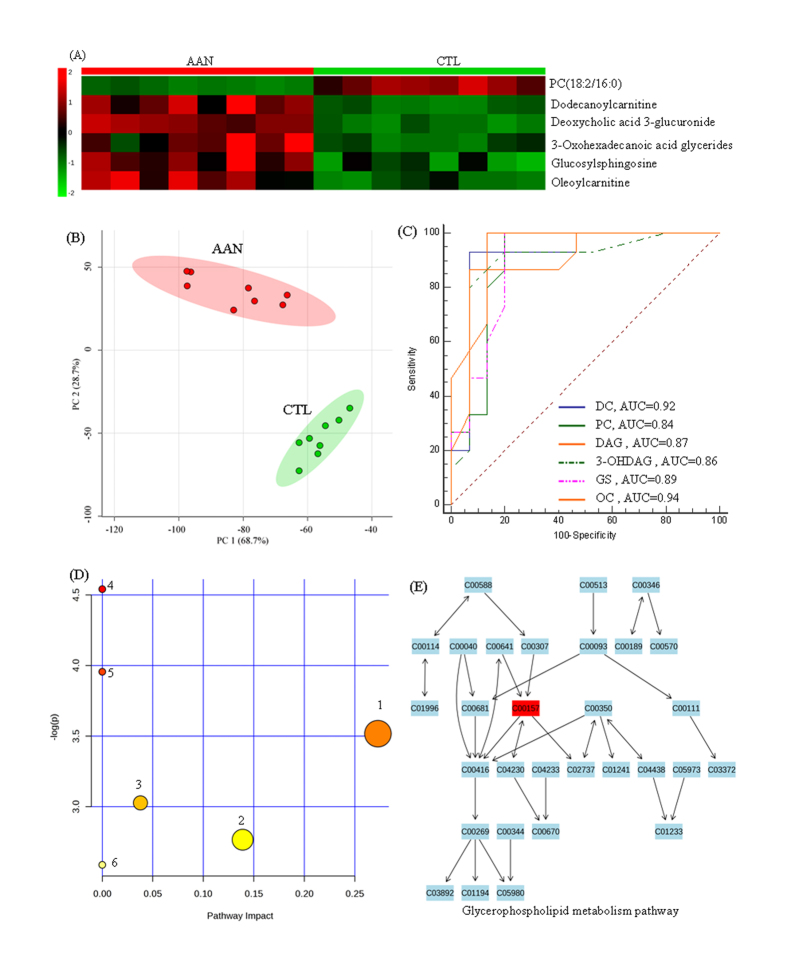
Hierarchical cluster analysis, ROC curves and metabolic pathway of metabolites in advanced nephrotoxicity (24th week). (**A**) Clustered heat map of seven metabolites only appeared at week 24. These identified metabolites showed a response on advanced nephrotoxicity in kidney tissue. (**B**) Principal component analysis shows separation of AAN group (red) and control group (green). PC1 versus PC2 were shown distribution of grouped individuals. The AAN and control groups could be separated completely based on seven identified metabolites. (**C**) PLS-DA-based ROC curves for the individual metabolites with high sensitivity and specificity from six metabolites. (**D**) Summary of pathway analysis with Ingenuity Pathway Analysis based on KEGG database. All the matched pathways were presented as circles. The color and size of each circle was based on p-value and pathway impact value, respectively. (1) linoleic acid metabolism; (2) alpha-linolenic acid metabolism; (3) pentose and glucuronate interconversions; (4) starch and sucrose metabolism; (5) glycerophospholipid metabolism; (6) arachidonic acid metabolism. (**E**) Pathway of glycerophospholipid metabolism with Ingenuity Pathway Analysis based on KEGG database. Compound numbers were consistent with KEGG database. DC, dodecanoylcarnitine; PC, PC(18:2/16:0); DAG, deoxycholic acid 3-glucuronide; 3-OHDAG, 3-oxohexadecanoic acid glycerides; GS, glucosylsphingosine; OC, oleoylcarnitine.

Sixty-eight metabolites were identified in the AAN rats at week 24 including six metabolites that appeared only at this stage. To further select the potential biomarker, a heat map was constructed. Significant reductions were found in dodecanoylcarnitine, deoxycholic acid 3-glucuronide, 3-oxohexadecanoic acid glycerides, glucosylsphingosine, oleoylcarnitine and PC(18:2/16:0) in the heat map (Fig. 6A). PCA was performed to find the metabolites that can discriminate AAN from control rats. Fig. 6B shows PCA of seven metabolites that could completely separate AAN rats from the control rats. The suitability of the seven metabolites for use as markers of advanced AAN was explored by PLS-DA-based ROC curves which revealed that except for PC(18:2/16:0), the other five metabolites have high sensitivities and specificities with AUC of 0.84 or greater (Fig. 6C).

The Ingenuity Pathway Analysis (IPA) was used to identify the biochemical pathways responsible for the observed metabolic abnormalities. Biological pathway analysis uncovered that seven metabolites involved in pentose and glucuronate inter-conversions, starch and sucrose metabolism, glycerophospholipid metabolism and fatty acid metabolism are affected by AAI exposure (Fig. 6D). The detailed construction of the glycerophospholipid metabolism pathway with higher score is shown in Fig. 6E. The results indicated that these seven metabolites show significant perturbations in advanced nephrotoxicity and could contribute to the development of AAN.

### Abnormalities of metabolites in the intermediary and advanced AAN (within 4 to 24 weeks)

Thirty-three metabolites including fifteen phosphatidylcholines (PC), seven lysophosphatidylcholines (lysoPC), six triglycerides (TG), four lysophosphatidylethanolamines (lysoPE), three phosphatidylethanolamines (PE) and one ceramide (Cer) were significantly altered in AAN rats at 4 to 24 weeks. To compare the contribution of the 33 altered lipids to different stages of AAN, we performed unsupervised cluster analyses focusing on each individual lipid species that exhibited statistically significant differences. Tissue samples from AAN rats segregated into tight clusters at different time points (Fig. 3B).

To further select the potential role of these lipid species, we constructed heat map which provided the relative average concentrations of the selected 33 metabolites in the AAN rats (Fig. 3C). Several metabolites showed a characteristic trend of alterations that corresponded with different stages of AAN within 4 to 24 weeks with some metabolites declining while other rising in the AAN rats kidney tissues. To identify metabolites indicative of AAN, criteria were used for selecting metabolites that were unidirectional and significantly changed across the different stages. Based on the selection criteria, seventeen putative metabolites were identified in kidney tissue. Concentrations of five PC, one PE, four lysoPC, four lysoPE and three TG steadily decreased from week 4 to 24. These results were consistent with their relative intensities (Fig. S6). To reduce the scope of the biomarkers, Hierarchical cluster analysis was employed to illuminate the potential relationships among the metabolites within 4 to 24 weeks. Accordingly, the identified 33 metabolites were clustered based on their Pearson correlation coefficients, which were indicated on the plot with different collars. The closely related metabolites were located in four major clusters including 11 PC, 4 TG, 4 lysoPC and 4 lysoPE (Fig. 3D).

According to the above results, the representative metabolites were selected from each cluster including five PC(14:1/20:1, 18:3/20:3, 20:4/p-16:0, 20:5/18:1, 22:4/14:0), four lysoPE(0:0/20:4, 0:0/22:5, 20:2/0:0, 20:3/0:0), three TG(14:0/14:0/14:1, 14:0/20:5/14:1, 18:4/22:6/22:6), two lysoPC(18:1, 20:4) and one PE(18:1/20:3). The representative metabolites in the same cluster also had similar alteration trends except for TG(14:0/14:0/14:1). Fig. S6 showed the changes of these characteristic metabolites from early and intermediary to advanced stages of nephropathy. Clear distinctions were observed in the abundance of individual metabolites which can serve as markers of different stages of AAN.

## Discussion

Identification of metabolic disorders and physiopathological mechanisms in the early and advanced stages of the disease are critical steps for prevention and treatment of nephrotoxicity including AAN which is a rapidly progressive tubulo-interstitial nephropathy. The rats with AAI-induced nephropathy exhibited marked polyuria, azotemia, anemia, and minimal proteinuria within 8 to 24 weeks. The histological examination of the kidney tissue showed intense tubulo-interstitial injury marked by heavy inflammatory cell infiltration, tubular dilation, and interstitial fibrosis within 8 to 24 weeks. This was accompanied by activation of the oxidative and inflammatory pathways as evidenced by increased p-IκB, and nuclear translocation of p65 along with up-regulation of pro-inflammatory and pro-oxidant molecules [NAD(P)H oxidase as well as COX-2, iNOS, MCP-1 and LOX-1]. Significant up-regulation of oxidative and inflammatory pathways was accompanied by severe impairment of Nrf2 activity as evidenced by the reduction in its nuclear content, elevation of its suppressor molecule, Keap1, and down-regulation of its target gene products including HO-1, NQO1, GCLC, GCLM, Cu,Zn-SOD, Mn-SOD and catalase. Renal interstitial fibrosis was associated with upregulation of pro-fibrotic mediators including Collagen I, TGF**-**β1, fibronectin and CD40, and down-regulation of anti-fibrosis factor including HGF and MMP-9. Activation of inflammatory, pro-oxidant, and fibrotic pathways and impairment of the Nrf2 system shown here are consistent with findings in other models of chronic kidney disease^9^,^10^,^11^. The impairment of renal function and structure in our AAN animals was accompanied by profound perturbation of kidney tissue lipid metabolism in early, intermediate and advanced phases of the disease. These findings are indicative of the potential role of lipid mediators in initiation, development, and progression of AAN as shown in previous studies^12^,^13^. The disturbances of glycerophospholipid, glycerolipid, prenol lipid, and sphingolipid metabolism and fatty acid oxidation, as well as bile acid biosynthesis were observed in the kidney tissue at various stages of AAN in rats employed in the present study. Changes in lipids including PC, TG, PE, lysoPE and lysoPC were closely association with histopathological abnormalities within weeks 4 to 24.

The underlying mechanism(s) responsible for the observed changes in different lipid metabolites in the kidney tissues of rats with AAN is presently unclear. One of the striking findings was reduction of PC and elevation of lysoPC which is most likely due to activation of phospholipase A2. Phospholipase A2 converts PC to lysoPC by catalysing removal of arachidonic acid from the sn-2 position of PC^14^,^15^,^16^. Release of arachidonic acid and its conversion to pro-inflammatory prostanoids by COX-2 represent an important step in promoting inflammation. In fact upregulation of COX-2 found here is consistently found in animal models of tubulointerstitial nephritis and glomerulosclerosis^9^,^10^,^11^. In addition, phospholipase-A2 catalyzes conversion of phosphatidylethanolamine which is a cell membrane component to lysophosphatidylethanolamine (LPE) by removing one of its fatty acid groups. Thus activation of phospholipase A2 can account for elevation of LPE and depression of PE in the AAN rats’ kidney tissues^17^. In fact a previous study has shown that LysoPC content correlates with phospholipase A2, and is significantly elevated in patients with diabetic nephropathy^18^. Changes in the lipid metabolism have been also found in the kidney of rats with adenine-induced chronic interstitial nephropathy^19^,^20^. Moreover, changes of triglyceride and ceramide have been demonstrated in numerous cases of herbal and diabetic nephropathies^5^,^15^. A number of previous studies have reported the association of abnormal lipid metabolism with activation of oxidative and inflammatory pathways and impairment of Nrf2 activity^21^,^22^. One study demonstrated that abnormal lipid metabolism was related to increased ROS production, NF-κB activation, impaired Nrf2 activation, glutathione depletion, inflammatory cell infiltration and fatty degeneration in the kidneys of mice with TiO_2_ nanoparticles-induced chronic kidney disease^23^.

PLS-DA-based ROC curves employed in the present study showed that six metabolites including two PC, two DG and two acyl carnitines were significantly altered prior to detectable changes in conventional chemical markers or kidney histology. Therefore they may be suitable metabolites for detection of AAI nephrotoxicity. Similarly, changes in eight metabolites including PC, TG, lysoPC, phytosphingosine and fatty acids showed high sensitivity and specificity in predicting the extent and severity of histopathological lesions, renal dysfunction, and activation of inflammatory, oxidative, and fibrotic pathways and impairment of the Nrf2 system. In this context, five metabolites including dodecanoylcarnitine, deoxycholic acid 3-glucuronide, 3-oxohexadecanoic acid glycerides, glucosylsphingosine and oleoylcarnitine showed high sensitivity and specificity as markers of advanced AAN. Changes in the above-mentioned metabolites indicated participation of several pathways of lipid metabolism involving fatty acids, bile acids, glycerolipids and phospholipids at different stages of AAN. In this context perturbations of fatty acid, phospholipid and glycerolipid metabolisms were found in early, intermediary, and advanced stages of AAN.

Fatty acid concentration in the kidney tissues of our AAN rats was significantly increased. This phenomenon is largely due to the recently demonstrated impairment of fatty acid oxidation in the tubular epithelial cells from animal models and humans with interstitial fibrosis^24^. The impairment of fatty acid oxidation limits mitochondrial ATP generation and enhances ROS production, events that contribute to tubular epithelial cell death, oxidative stress, inflammation and interstitial fibrosis.

The tissue contents of two acyl carnitines were significantly reduced in the kidneys of our AAN rats. Delivery of fatty acids to the mitochondria requires their binding to L-carnitine a process that is catalysed by the enzyme, carnitine palmitoyltransferase 1^25^. Acyl-carnitines including stearoylcarnitine, L-palmitoylcarnitine, oleoylcarnitine and dodecanoylcarnitine are essential for delivery of lipid fuel for production of energy by mitochondria and therefore cell growth and survival^25^. The kidney tissues from our AAN rats showed significant increase in diacylglycerols including DG(15:0/20:5/0:0) and DG(15:0/0:0/15:0). Diacylglycerols are substrates for formation of triglycerides by the enzyme diacylglycerol acyltransferase (DGAT). A previous study by our group has shown down-regulation of DGAT in the liver of rats with chronic renal failure^26^. The CKD associated down-regulation of DGAT can account for the observed accumulation of diacylglycerol in our CKD rats with AAN. Accumulation of diacylglycerol has been shown to cause protein kinase C activation, which contributes to the initiation and development of diabetic nephropathy^27^. Protein kinase C promotes activation of NF-κB and upregulation of TGF-β1, plasminogen activator inhibitor, vascular endothelial growth factor and NADHP oxidase events that lead to mesangial expansion, interstitial fibrosis, and oxidative stress^27^. This study has some limitations. I- the underlying mechanism by which AAI exposure alters lipid metabolism in the renal tissue is presently unclear and requires further investigation, II- age and gender can potentially impact the susceptibility to renal injury which was not addressed in the present study. III- The metabolomic abnormalities found in the AAN model of chronic interstitial nephropathy may not reflect those associated with other forms of chronic kidney disease in humans and animals.

In conclusion, the present study revealed the link between activations of oxidative, inflammatory, and fibrotic pathways and dynamic changes in fatty acid, phospholipid, and glycerolipid metabolisms in AAN. Significant changes were observed in several metabolites prior to the onset of detectable abnormalities of kidney function or histology. In addition profound changes in fatty acid and phospholipid metabolites were observed in the intermediary and advanced stages of AAN. These findings demonstrate the participation of altered tissue lipid metabolism in the pathogenesis of AAI nephrotoxicity.

## Methods

### Animals

Nine week-old male Sprague-Dawley rats, weighting 200 ± 10 g were purchased from Fourth Military Medical University (Xi’an, China). The rats were randomized to the AAN (n = 40) and control groups (n = 40). The AAN group was orally administered 20 mg/kg body weight/week of AAI in 0.5% NaHCO_3_ solution by oral gavage for 12 weeks and observed for an additional 12 weeks. Aristolochic acid-I was obtained from the National Institutes for Food and Drug Control (Beijing, China). The control group was treated with the vehicle instead. At weeks 0, 4, 8, 12 and 24, eight rats from each group were selected randomly and placed in metabolic cages to obtain 24 h urine collections. They were then anesthetized with 10% urethane, blood samples were obtained by carotid artery cannula, and kidneys were immediately removed and processed for histological evaluation, Western blotting, and metabolomics analyses. Blood and urine samples were centrifuged at 3000 rpm for 10 min and the supernatants were collected and stored at –80 °C. The study was approved by the Ethical Committee of Northwest University, and all procedures were in accordance with the Helsinki Declaration.

### Biochemical determination

Plasma and urine biochemistry were analyzed as described in detail previously^28^. Serum malondialdehyde (MDA), renal thiobarbituric acid-reactive substance (TBARS), oxidized glutathione (GSSG) and reduced glutathione (GSH) were measured as described in detail previously^29^. Osmolality was measured by the freezing point depression method using Advanced Osmometer. Urine pH was measured using pH meter.

### Histological evaluation and Western blot analysis

Kidneys were fixed in 10% buffered formalin and embedded in Paraffin. H&E staining, Masson staining and immunohistochemisrty were performed as described in detail previously^7^,^30^, and Morphometric quantification were conducted using the ImageJ software. All the antibodies were purchased from Santa Cruz Biotechnology or Abcam Company. All the Western blot analyses were performed as described previously^9^.

### Sample preparation and UPLC-MS analysis

Kidney tissue samples were prepared as described previously^7^. Metabolomics was performed on a Waters Acquity^TM^ Ultra Performance LC system equipped with a Waters Xevo^TM^ G2-S QTof MS. chromatographic separation, mass spectrometry were described in detail in the Supplementary Methods online. Figure S7 shows a typical example of the workflow of metabolomics using UPLC Q-TOF/MS as a research tools for discovering and quantifying metabolites in complex mixtures.

### Statistical analysis

Metabolites were identified from loading plots and S-plots of orthogonal partial least squares discriminant analysis (OPLS-DA) using the Markerlynx XS and Progenesis QI (Waters Corporation, MA, USA). Additional statistical analyses were performed using SPSS 16.0, Metaboanalysis 3.0 and MedCalc 13.0. Metabolite differences were considered significant when test *P* values were less than 0.05.

## Additional Information

**How to cite this article**: Zhao, Y.-Y. *et al.* Metabolomics analysis reveals the association between lipid abnormalities and oxidative stress, inflammation, fibrosis, and Nrf2 dysfunction in aristolochic acid-induced nephropathy. *Sci. Rep.* **5**, 12936; doi: 10.1038/srep12936 (2015).

## Supplementary Material

Supplementary Information

## Figures and Tables

**Table 1:** General parameters.

Parameters	Group	0 wk	4 wk	8 wk	12 wk	24 wk
Body weight (g)	CTL	201.9 ± 10.2	319.5 ± 21.6	393.0 ± 32.8	437.0 ± 33.4	565.5 ± 48.7
	Model	187.8 ± 10.7	279.7 ± 20.4**	329.2 ± 26.7**	354.7 ± 32.2**	378.3 ± 42.5**
Urine volume (ml)	CTL	6.85 ± 1.21	8.15 ± 2.14	11.55 ± 2.35	13.06 ± 2.45	14.95 ± 2.15
	Model	7.02 ± 0.98	11.56 ± 2.98*	14.25 ± 1.95*	16.11 ± 3.02*	18.12 ± 3.18*
Urine specific gravity	CTL	1.045 ± 0.006	1.052 ± 0.009	1.048 ± 0.008	1.047 ± 0.012	1.063 ± 0.013
	Model	1.049 ± 0.009	1.048 ± 0.007	1.033 ± 0.015*	1.009 ± 0.009**	1.002 ± 0.014**
Urine pH	CTL	6.82 ± 0.21	6.45 ± 0.25	7.04 ± 0.17	6.85 ± 0.23	7.12 ± 0.19
	Model	6.79 ± 0.13	6.76 ± 0.18*	7.95 ± 0.41**	8.24 ± 0.55**	8.03 ± 0.48**
Urine osmolality (Mosm/kg·H_2_O)	CTL	1632 ± 119	1650.7 ± 146	1694.7 ± 158	1689 ± 125	1725 ± 156
	Model	1615 ± 153.7	1568 ± 128	1389 ± 165**	1178 ± 187**	1008 ± 217**
Urine protein (mg/day)	CTL	8.30 ± 2.68	9.59 ± 3.12	9.28 ± 4.25	11.89 ± 3.95	10.44 ± 3.15
	Model	8.52 ± 3.62	10.56 ± 4.04	19.34 ± 7.56**	31.78 ± 8.56**	38.05 ± 9.11**
Urine creatinine (mmol/l)	CTL	11543 ± 1598	11438 ± 1962	9261 ± 1999	10258 ± 2077	9586 ± 1862
	Model	11157 ± 1251	8922 ± 2383	5217 ± 1082**	4723 ± 1255**	3483 ± 1519**
Urine sodium (mmol/l)	CTL	42.2 ± 5.6	40.6 ± 4.6	42.5 ± 7.9	40.8 ± 6.7	45.9 ± 5.9
	Model	41.5 ± 4.9	52.8 ± 15*	54.8 ± 10.5*	59.6 ± 12.5**	63.7 ± 15.9*
Serum sodium (mmol/l)	CTL	141.2 ± 1.2	144.5 ± 3.2	142.7 ± 2.7	141.7 ± 3.2	143.4 ± 2.3
	Model	140.8 ± 1.4	142.4 ± 2.7	147.2 ± 8.6	150.3 ± 9.1	148.4 ± 7.5
Serum MDA (μmol/l)	CTL	0.23 ± 0.09	0.25 ± 0.11	0.32 ± 0.07	0.26 ± 0.09	0.30 ± 0.13
	Model	0.25 ± 0.08	0.29 ± 0.13	1.02 ± 0.31**	1.74 ± 0.52**	2.82 ± 0.65**
Kidney TBARS (mmol/kg protein)	CTL	0.24 ± 0.09	0.29 ± 0.08	0.26 ± 0.06	0.29 ± 0.12	0.35 ± 0.15
	Model	0.21 ± 0.08	0.25 ± 0.09	0.36 ± 0.09*	0.42 ± 0.15**	0.51 ± 0.25**
Reduced GSH/GSSG ratio	CTL	5.05 ± 1.32	4.98 ± 1.22	5.54 ± 1.34	5.35 ± 1.07	5.68 ± 1.89
	Model	5.62 ± 1.24	5.35 ± 1.42	4.03 ± 0.96*	3.05 ± 1.26**	2.48 ± 0.84**
Mitochondrial TBABS (mmol/kg protein)	CTL	0.38 ± 0.14	0.40 ± 0.17	0.35 ± 0.13	0.43 ± 0.18	0.45 ± 0.26
	Model	0.35 ± 0.11	0.43 ± 0.21	0.59 ± 0.23*	0.85 ± 0.25**	1.03 ± 0.36**

Results are expressed as the means ± standard deviation, *P < 0.05, **P < 0.01 compared with control rats by two-way between-group ANOVA.

**Table 2:** Differential metabolites and their sensitivities and specificities for discrimination between AAN and control rats in the 4th week.

Metabolites	AUC	95%CI	Sensitivity (%)	Specificity (%)
**PC(18:3)**	**0.95**	**0.81**–**0.99**	**94.3**	**93.7**
**DG(15:0/20:5/0:0)**	**0.94**	**0.84**–**0.98**	**86.3**	**87.5**
**DG(15:0/0:0/15:0)**	**0.92**	**0.84**–**0.98**	**93.4**	**85.1**
**Stearoylcarnitine**	**0.89**	**0.82**–**0.95**	**86.3**	**93.7**
**Palmitoylcarnitine**	**0.88**	**0.81**–**0.98**	**93.3**	**87.7**
TG(14:1/18:1/14:1)	0.86	0.73–0.92	72.3	77.5
TG(14:0/14:0/14:0)	0.85	0.70–0.92	83.2	62.5
**PC(18:3/22:6)**	**0.85**	**0.73**–**0.91**	**86.6**	**89.3**
PE(18:4/22:4)	0.85	0.74–0.91	86.7	65.9
PC(16:1/18:1)	0.84	0.81–0.95	69.7	93.7
PC(20:1/18:3)	0.78	0.61–0.88	83.3	57.5
Arachidic acid	0.77	0.88–1.00	82.3	63.7
PE(20:3/P-18:1)	0.74	0.55–0.88	83.3	62.5
LysoPE(18:0/0:0)	0.74	0.51–0.86	63.3	87.5
Docosahexaenoic acid	0.73	0.63–0.89	62.3	81.2
PC(22:6/16:1)	0.72	0.64–0.83	71.3	87.5
PE(20:0/18:0)	0.72	0.53–0.88	66.7	81.2
LysoPE(0:0/16:0)	0.68	0.55–0.79	69.5	73.1
Eicosatetraenoic acid	0.67	0.44–0.79	71.1	61.2
PC(16:1/22:6)	0.66	0.47–0.82	85.3	62.5
PC(20:4/14:0)	0.65	0.49–0.85	74.3	83.7
PE(22:5/20:3)	0.62	0.41–0.72	56.8	67.6
