# Supplementary material for: Metabolomics analysis reveals the association between lipid abnormalities and oxidative stress, inflammation, fibrosis, and Nrf2 dysfunction in aristolochic acid-induced nephropathy
Source: Sci Rep. 2015 Aug 7;5:12936. doi: 10.1038/srep12936 (PMC4528220; doi:10.1038/srep12936)
Supplement: Supplementary Information [file srep12936-s1.doc]

**Metabolomics analysis reveals the association between lipid abnormalities and oxidative stress, inflammation, fibrosis, and Nrf2 dysfunction in aristolochic acid-induced nephropathy**

Ying-Yong Zhao^a,b*^, Hui-Ling Wang^f^, Xian-Long Cheng^d^, Feng Wei^d^, Xu Bai^e^, Rui-Chao Lin^c^, Nosratola D Vaziri^b,*^

^a^ Key Laboratory of Resource Biology and Biotechnology in Western China, Ministry of Education, the College of Life Sciences, Northwest University, No. 229 Taibai North Road, Xi’an, Shaanxi 710069, China Division of

^b^ Division of Nephrology and Hypertension, School of Medicine, University of California, Irvine, MedSci 1, C352, UCI Campus, Irvine, California, 92897, USA

^c^ School of Chinese Materia Medica, Beijing University of Chinese Medicine, No. 11 North Third Ring Road, Beijing 100029, China

^d^ National Institutes for Food and Drug Control, State Food and Drug Administration, No. 2 Tiantan Xili, Beijing, 100050, China

^e^ Solution Centre, Waters Technologies (Shanghai) Ltd., No. 1000 Jinhai Road, Shanghai 201203, China

^f^ Department of nephrology, Shanghai Jimin Hospital, No. 338 Huaihai West Road, Shanghai 200052, China

**SUPPLEMENTARY MATERIAL**

**Chromatographic separation of metabolomics analysis**

The UPLC analysis was performed on a 2.1 mm × 100 mm ACQUITY 1.8 µm HSS T3 using a Waters Acquity^TM^ UPLC system equipped with a Waters Xevo^TM^ G2 QTof MS. A gradient of 0.1% formic acid in acetonitrile (A) and 0.1% formic acid in water (B) used as follows: a linear gradient of 0–2.0 min, 1–60% A; 2.0–6.0 min, 60–85% A; 6.0–8.0 min, 85-99% A; 8.0–10.0 min, 99-1% and 10.0–13.0 min, 1%. The flow rate was 0.45 ml/min. The autosampler was maintained at 4 °C. Every 2 µL sample solution was injected for each run.

Mass spectrometry of the optimal conditions were as follows: capillary voltage of 1.5 kV for negative ion mode, cone voltage of 15 V, desolvation gas temperature of 450 °C, source temperature of 120 °C, desolvation gas flow of 700 L/h, cone gas flow of 30 L/h, The scan range was from 50 to 1000 m/z. Leucine–enkephalin was used for accurate mass acquisition. Waters MassLynx v4.1 was used for all the acquisition and analysis of data.

**Data processing and standardization metabolites**

The raw data were analyzed using the Applied Waters Markerlynx XS software, this allowed deconvolution, alignment and data reduction to give a list of mass and retention time pairs with corresponding intensities for all the detected peaks from each data file in the data set. The main parameters were set as follows: retention time range 1– 10 min, mass range 50–1200 amu, mass tolerance 0.01, minimum intensity 1%, mass window 0.05, retention time window 0.20, and noise elimination level 6. All of the data were normalized to the summed total ion intensity per chromatogram, and the resultant data matrices were introduced to the EZinfo 2.0 software for principal component analysis (PCA) and orthogonal partial least squares discriminant analysis (OPLS-DA).

Table S1

Identified biomarkers of AAN detected by UPLC-MS in positive ionization mode.

| No. | m/z | Lipid | Class | No. | m/z | Lipid | Class |
| --- | --- | --- | --- | --- | --- | --- | --- |
| 4 wk |  |  |  |  |  |  |  |
| 1 | 758.5752 | PC(14:1/20:1) | GP | 37 | 732.5599 | DG(22:5/0:0/22:6) | GL |
| 2 | 806.5747 | PC(20:5/18:1) | GP | 38 | 329.2471 | Docosahexaenoic acid | FA |
| 3 | 806.5753 | PC(18:3/20:3) | GP | 39 | 502.2986 | LysoPE(0:0/20:4) | GP |
| 4 | 760.5904 | PC(18:1/16:0) | GP | 40 | 993.6904 | TG(18:4/22:6/22:6) | GL |
| 5 | 522.3557 | LysoPC(18:1) | GP | 41 | 438.2964 | Palmitoylcarnitine | FA |
| 6 | 780.5592 | PC(18:3) | GP | 42 | 811.6177 | TG(14:1/18:1/14:1) | GL |
| 7 | 544.3405 | LysoPC(20:4) | GP | 43 | 767.5850 | TG(14:1/18:4/14:1) | GL |
| 8 | 832.5909 | PC(18:1/22:6) | GP | 44 | 339.2887 | 5,6-DHET | FA |
| 9 | 991.6809 | TG(22:4/20:4/18:3) | GL | 45 | 454.2919 | LysoPE(0:0/16:0) | GP |
| 10 | 780.5569 | PC(16:1/20:4) | GP | 46 | 524.3715 | LysoPC(18:0) | GP |
| 11 | 758.5713 | PC(16:1/18:1) | GP | 47 | 804.5545 | PC(16:1/22:6) | GP |
| 12 | 810.6099 | PC(20:1/18:3) | GP | 48 | 789.5669 | DG(24:1/22:6/0:0) | GL |
| 13 | 804.5549 | PC(18:3/20:4) | GP | 49 | 852.5577 | PC(20:5/20:3) | GP |
| 14 | 759.5800 | TG(14:0/14:0/14:1) | GL | 50 | 828.5545 | PC(20:4/20:5) | GP |
| 15 | 833.5956 | TG(14:0/20:5/14:1) | GL | 51 | 521.3460 | LysoPE(20:3/0:0) | GP |
| 16 | 805.5586 | PE(18:3/22:5) | GP | 52 | 963.6486 | TG(22:5/14:1/22:5) | GL |
| 17 | 768.5648 | PE(18:1/20:3) | GP | 53 | 834.6042 | PC(18:1/22:5) | GP |
| 18 | 568.3416 | LysoPC(22:6) | GP | 54 | 784.5883 | PC(22:2/14:1) | GP |
| 19 | 807.5794 | TG(14:1/14:1/18:3) | GL | 55 | 313.2718 | Arachidic acid | FA |
| 20 | 520.3412 | LysoPC(18:2) | GP | 56 | 305.2482 | Eicosatetraenoic acid | FA |
| 21 | 761.5977 | TG(14:0/14:0/14:0) | GL | 57 | 907.5846 | TG(18:4/18:3/18:4) | GL |
| 22 | 782.5739 | PC(22:4/14:0) | GP | 58 | 959.6155 | TG(18:4/20:4/20:5) | GL |
| 23 | 766.5792 | PC(20:4/P-16:0) | GP | 59 | 820.5300 | PC(20:3/16:1) | GP |
| 24 | 545.3455 | LysoPE(0:0/22:5) | GP | 60 | 804.5519 | PC(22:6/16:1) | GP |
| 25 | 523.3606 | LysoPE(20:2/0:0) | GP | 61 | 814.5874 | PE(20:0/18:0) | GP |
| 26 | 781.5631 | PE(18:3/20:3) | GP | 62 | 623.5054 | DG(15:0/20:5/0:0) | GL |
| 27 | 788.5705 | PC(P-18:1/18:3) | GP | 63 | 428.3738 | Stearoylcarnitine | FA |
| 28 | 830.5746 | PC(20:4/20:4) | GP | 64 | 810.6078 | PC(20:4/18:0) | GP |
| 29 | 784.5928 | PC(18:3/18:0) | GP | 65 | 782.5693 | PC(20:4/16:0) | GP |
| 30 | 834.6005 | PC(22:5/18:1) | GP | 66 | 792.5813 | PC(20:4/P-18:1) | GP |
| 31 | 769.5704 | PE(20:3/P-18:1) | GP | 67 | 783.5789 | TG(14:1/16:1/14:1) | GL |
| 32 | 558.4870 | DG(15:0/0:0/15:0) | GL | 68 | 754.5382 | PC(20:4/14:0) | GP |
| 33 | 480.3329 | LysoPC(P-16:0) | GP | 69 | 540.5350 | Cer(d18:0/16:0) | SL |
| 34 | 482.3226 | LysoPE(18:0/0:0) | GP | 70 | 767.5831 | DG(24:0/20:4/0:0) | GL |
| 35 | 810.6073 | PE(18:4/22:4) | GP | 71 | 833.5929 | PE(22:5/20:3) | GP |
| 36 | 546.3558 | LysoPC(20:3) | GP | 72 | 828.5575 | PC(18:3/22:6) | GP |
| 8 wk |  |  |  |  |  |  |  |
| 1 | 758.5752 | PC(14:1/20:1) | GP | 36 | 828.5575 | PC(18:3/22:6) | GP |
| 2 | 522.3557 | LysoPC(18:1) | GP | 37 | 781.5635 | PE(18:3/20:3) | GP |
| 3 | 544.3405 | LysoPC(20:4) | GP | 38 | 804.5552 | PC(18:2/20:5) | GP |
| 4 | 806.5747 | PC(20:5/18:1) | GP | 39 | 547.3602 | LysoPE(22:4/0:0) | GP |
| 5 | 806.5753 | PC(18:3/20:3) | GP | 40 | 339.2887 | 5,6-DHET | FA |
| 6 | 991.6809 | TG(22:4/20:4/18:3) | GL | 41 | 769.5704 | PE(20:3/P-18:1) | GP |
| 7 | 759.5800 | TG(14:0/14:0/14:1) | GL | 42 | 817.6145 | TG(14:0/14:1/20:5) | GL |
| 8 | 520.3412 | LysoPC(18:2) | GP | 43 | 545.3460 | LysoPE(22:5/0:0) | GP |
| 9 | 780.5592 | PC(18:2/18:3) | GP | 44 | 788.5705 | PC(P-18:1/18:3) | GP |
| 10 | 568.3416 | LysoPC(22:6) | GP | 45 | 524.3715 | LysoPC(18:0) | GP |
| 11 | 804.5549 | PC(18:3/20:4) | GP | 46 | 497.3446 | LysoPE(18:1/0:0) | GP |
| 12 | 545.3455 | LysoPE(0:0/22:5) | GP | 47 | 807.5801 | TG(14:1/18:3/14:1) | GL |
| 13 | 810.6073 | PE(18:4/22:4) | GP | 48 | 808.5886 | PC(16:0/22:5) | GP |
| 14 | 523.3606 | LysoPE(20:2/0:0) | GP | 49 | 305.2482 | Arachidonic acid | FA |
| 15 | 546.3558 | LysoPC(20:3) | GP | 50 | 828.5545 | PC(20:4/20:5) | GP |
| 16 | 760.5904 | PC(18:1/16:0) | GP | 51 | 780.5569 | PC(16:1/20:4) | GP |
| 17 | 782.5739 | PC(22:4/14:0) | GP | 52 | 400.3416 | Palmitoylcarnitine | FA |
| 18 | 780.5582 | PC(22:5/14:0) | GP | 53 | 757.5631 | TG(14:1/14:0/14:1) | GL |
| 19 | 810.6078 | PC(20:4/18:0) | GP | 54 | 834.6005 | PC(22:5/18:1) | GP |
| 20 | 496.3393 | LysoPC(16:0) | GP | 55 | 318.2987 | Phytosphingosine | SL |
| 21 | 832.5909 | PC(18:1/22:6) | GP | 56 | 540.5350 | Cer(d18:0/16:0) | SL |
| 22 | 805.5586 | PE(18:3/22:5) | GP | 57 | 766.5792 | PC(20:4/P-16:0) | GP |
| 23 | 768.5648 | PE(18:1/20:3) | GP | 58 | 783.5789 | TG(14:1/16:1/14:1) | GL |
| 24 | 274.2724 | Trimethyltridecanoic acid | PL | 59 | 833.5929 | PE(22:5/20:3) | GP |
| 25 | 480.3329 | LysoPC(P-16:0) | GP | 60 | 834.6042 | PC(18:1/22:5) | GP |
| 26 | 807.5794 | TG(14:1/14:1/18:3) | GL | 61 | 784.5883 | PC(22:2/14:1) | GP |
| 27 | 521.3460 | LysoPE(20:3/0:0) | GP | 62 | 512.5030 | Cer(d18:0/14:0) | SL |
| 28 | 502.2986 | LysoPE(0:0/20:4) | GP | 63 | 820.5300 | PC(20:3/16:1) | GP |
| 29 | 830.5746 | PC(20:4/20:4) | GP | 64 | 818.6198 | PC(22:5/P-18:1) | GP |
| 30 | 810.6099 | PC(20:1/18:3) | GP | 65 | 782.5693 | PC(20:4/16:0) | GP |
| 31 | 361.2736 | Tetracosatetraenoic acid | FA | 66 | 852.5577 | PC(20:5/20:3) | GP |
| 32 | 833.5956 | TG(14:0/20:5/14:1) | GL | 67 | 758.5713 | PC(16:1/18:1) | GP |
| 33 | 993.6904 | TG(18:4/22:6/22:6) | GL | 68 | 521.3457 | Acetylursolic acid | FA |
| 34 | 784.5928 | PC(18:3/18:0) | GP | 69 | 959.6155 | TG(18:4/20:4/20:5) | GL |
| 35 | 756.5530 | PC(18:2/16:1) | GP | 70 |  |  |  |
| 12 wk |  |  |  |  |  |  |  |
| 1 | 544.3405 | LysoPC(20:4) | GP | 33 | 521.3457 | Acetylursolic acid | FA |
| 2 | 758.5752 | PC(14:1/20:1) | GP | 34 | 769.5704 | PE(20:3/P-18:1) | GP |
| 3 | 522.3557 | LysoPC(18:1) | GP | 35 | 833.5956 | TG(14:0/20:5/14:1) | GL |
| 4 | 568.3416 | LysoPC(22:6) | GP | 36 | 766.5792 | PC(20:4/P-16:0) | GP |
| 5 | 806.5747 | PC(20:5/18:1) | GP | 37 | 512.5030 | Cer(d18:0/14:0) | SL |
| 6 | 520.3412 | LysoPC(18:2) | GP | 38 | 540.5350 | Cer(d18:0/16:0) | SL |
| 7 | 759.5800 | TG(14:0/14:0/14:1) | GL | 39 | 570.3578 | LysoPC(22:5) | GP |
| 8 | 806.5753 | PC(18:3/20:3) | GP | 40 | 781.5635 | PE(18:3/20:3) | GP |
| 9 | 546.3558 | LysoPC(20:3) | GP | 41 | 828.5545 | PC(20:4/20:5) | GP |
| 10 | 545.3455 | LysoPE(0:0/22:5) | GP | 42 | 447.2495 | Cholic acid | ST |
| 11 | 780.5592 | PC(18:2/18:3) | GP | 43 | 305.2482 | Arachidonic acid | FA |
| 12 | 804.5549 | PC(18:3/20:4) | GP | 44 | 757.5631 | TG(14:1/14:0/14:1) | GL |
| 13 | 991.6809 | TG(22:4/20:4/18:3) | GL | 45 | 782.5693 | PC(20:4/16:0) | GP |
| 14 | 523.3606 | LysoPE(20:2/0:0) | GP | 46 | 760.5904 | PC(18:1/16:0) | GP |
| 15 | 810.6078 | PC(20:4/18:0) | GP | 47 | 400.3416 | Palmitoylcarnitine | FA |
| 16 | 782.5739 | PC(22:4/14:0) | GP | 48 | 524.3641 | LysoPC(18:0) | GP |
| 17 | 768.5648 | PE(18:1/20:3) | GP | 49 | 542.3243 | LysoPC(20:5) | GP |
| 18 | 810.6073 | PE(18:4/22:4) | GP | 50 | 808.5886 | PC(16:0/22:5) | GP |
| 19 | 521.3460 | LysoPE(20:3/0:0) | GP | 51 | 807.5801 | TG(14:1/18:3/14:1) | GL |
| 20 | 805.5586 | PE(18:3/22:5) | GP | 52 | 784.5883 | PC(22:2/14:1) | GP |
| 21 | 545.3460 | LysoPE(22:5/0:0) | GP | 53 | 494.3230 | LysoPC(16:1) | GP |
| 22 | 502.2986 | LysoPE(0:0/20:4) | GP | 54 | 548.3707 | LysoPC(20:2) | GP |
| 23 | 547.3602 | LysoPE(22:4/0:0) | GP | 55 | 993.6904 | TG(18:4/22:6/22:6) | GL |
| 24 | 496.3393 | LysoPC(16:0) | GP | 56 | 503.3010 | Crustecdysone | ST |
| 25 | 832.5909 | PC(18:1/22:6) | GP | 57 | 820.5300 | PC(20:3/16:1) | GP |
| 26 | 807.5794 | TG(14:1/14:1/18:3) | GL | 58 | 761.5977 | TG(14:0/14:0/14:0) | GL |
| 27 | 784.5928 | PC(18:3/18:0) | GP | 59 | 834.6005 | PC(22:5/18:1) | GP |
| 28 | 804.5552 | PC(18:2/20:5) | GP | 60 | 830.5746 | PC(20:4/20:4) | GP |
| 29 | 756.5530 | PC(18:2/16:1) | GP | 61 | 796.5969 | PE(20:3/20:1) | GP |
| 30 | 811.6126 | TG(14:1/18:1/14:1) | GL | 62 | 313.2739 | Arachidic acid | FA |
| 31 | 766.5798 | PC(P-18:1/18:3) | GP | 63 | 959.6155 | TG(18:4/20:4/20:5) | GL |
| 32 | 828.5575 | PC(18:3/22:6) | GP | 64 | 480.3329 | LysoPC(P-16:0) | GP |
| 24 wk |  |  |  |  |  |  |  |
| 1 | 758.5752 | PC(14:1/20:1) | GP | 35 | 502.2986 | LysoPE(0:0/20:4) | GP |
| 2 | 991.6809 | TG(22:4/20:4/18:3) | GL | 36 | 830.5746 | PC(20:4/20:4) | GP |
| 3 | 544.3405 | LysoPC(20:4) | GP | 37 | 784.5928 | PC(18:3/18:0) | GP |
| 4 | 806.5753 | PC(18:3/20:3) | GP | 38 | 817.6145 | TG(14:0/14:1/20:5) | GL |
| 5 | 759.5800 | TG(14:0/14:0/14:1) | GL | 39 | 545.3460 | LysoPE(22:5/0:0) | GP |
| 6 | 780.5592 | PC(18:2/18:3) | GP | 40 | 768.5648 | PE(18:1/20:3) | GP |
| 7 | 806.5747 | PC(20:5/18:1) | GP | 41 | 766.5798 | PC(P-18:1/18:3) | GP |
| 8 | 522.3557 | LysoPC(18:1) | GP | 42 | 810.6099 | PC(20:1/18:3) | GP |
| 9 | 496.3394 | LysoPC(16:0) | GP | 43 | 834.6005 | PC(22:5/18:1) | GP |
| 10 | 524.3715 | LysoPC(18:0) | GP | 44 | 828.5545 | PC(20:4/20:5) | GP |
| 11 | 758.5757 | PC(18:2/16:0) | GP | 45 | 757.5631 | TG(14:1/14:0/14:1) | GL |
| 12 | 832.5909 | PC(18:1/22:6) | GP | 46 | 480.3329 | LysoPC(P-16:0) | GP |
| 13 | 447.2495 | Cholic acid | ST | 47 | 454.2919 | LysoPE(0:0/16:0) | GP |
| 14 | 545.3455 | LysoPE(0:0/22:5) | GP | 48 | 959.6155 | TG(18:4/20:4/20:5) | GL |
| 15 | 760.5904 | PC(18:1/16:0) | GP | 49 | 540.5350 | Cer(d18:0/16:0) | SL |
| 16 | 810.6078 | PC(20:4/18:0) | GP | 50 | 807.5801 | TG(14:1/18:3/14:1) | GL |
| 17 | 361.2736 | Dodecanoylcarnitine | ST | 51 | 428.3738 | Stearoylcarnitine | FA |
| 18 | 782.5739 | PC(22:4/14:0) | GP | 52 | 808.5886 | PC(16:0/22:5) | GP |
| 19 | 520.3412 | LysoPC(18:2) | GP | 53 | 305.2482 | Eicosatetraenoic acid | FA |
| 20 | 804.5549 | PC(18:3/20:4) | GP | 54 | 784.5883 | PC(22:2/14:1) | GP |
| 21 | 568.3416 | LysoPC(22:6) | GP | 55 | 512.5030 | Cer(d18:0/14:0) | SL |
| 22 | 833.5956 | TG(14:0/20:5/14:1) | GL | 56 | 783.5789 | TG(14:1/16:1/14:1) | GL |
| 23 | 400.3416 | L-Palmitoylcarnitine | FA | 57 | 521.3460 | LysoPE(20:3/0:0) | GP |
| 24 | 766.5792 | PC(20:4/P-16:0) | GP | 58 | 758.5713 | PC(16:1/18:1) | GP |
| 25 | 313.2739 | Arachidic acid | FA | 59 | 569.3472 | Deoxycholic acid 3-glucuronide | ST |
| 26 | 756.5530 | PC(18:2/16:1) | GP | 60 | 362.2797 | 3-Oxohexadecanoic acid glycerides | FA |
| 27 | 993.6904 | TG(18:4/22:6/22:6) | GL | 61 | 341.3037 | Docosahexaenoic acid | FA |
| 28 | 339.2887 | 5,6-DHET | FA | 62 | 482.3226 | LysoPE(18:0/0:0) | GP |
| 29 | 804.5552 | PC(18:2/20:5) | GP | 63 | 546.3558 | LysoPC(20:3) | GP |
| 30 | 805.5586 | PE(18:3/22:5) | GP | 64 | 462.2968 | Glucosylsphingosine | SL |
| 31 | 523.3606 | LysoPE(20:2/0:0) | GP | 65 | 833.5929 | PE(22:5/20:3) | GP |
| 32 | 811.6126 | TG(14:1/18:1/14:1) | GL | 66 | 907.5846 | TG(18:4/18:3/18:4) | GL |
| 33 | 497.3446 | LysoPE(18:1/0:0) | GP | 67 | 818.6198 | PC(22:5/P-18:1) | GP |
| 34 | 781.5635 | PE(18:3/20:3) | GP | 68 | 426.3568 | Oleoylcarnitine | FA |

FA: fatty acyls; GL: glycerolipid; GP glycerophospholipid; PL: prenol lipid; SL: sphingolipid; ST: sterol lipid.


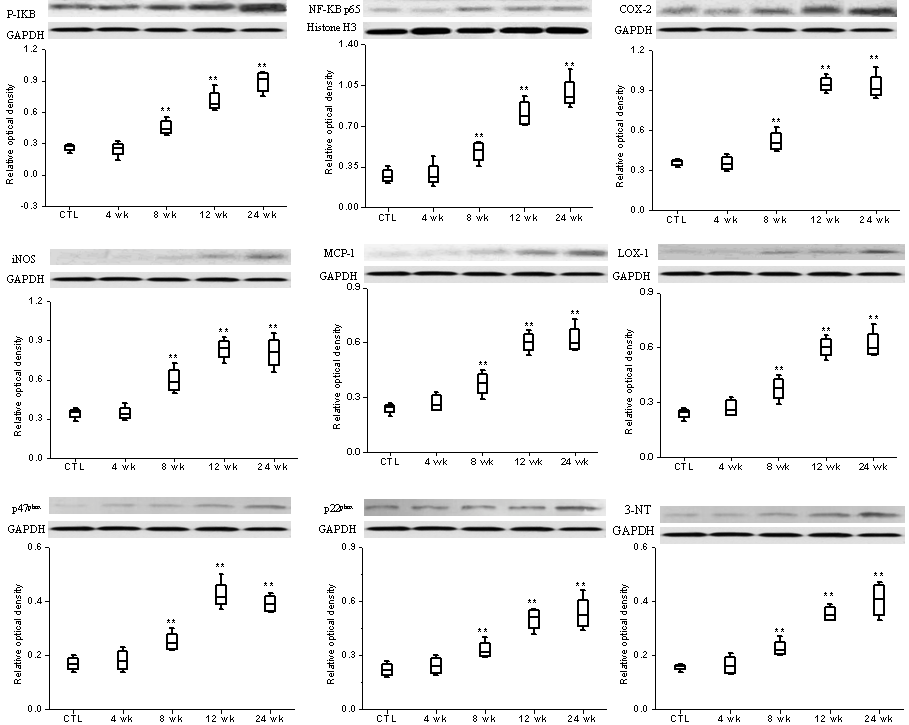


Figure S1 NF-κB target protein expressions in kidney from model rats. Representative Western blots depicting p-IκB and nuclear content of p65 active subunit of NF-κB as well as expression of COX-2, iNOS, MCP-1, LOX-1, NOX4, p47^phox^, p22^phox^ and 3-nitrotyrosine in the kidney tissues of the control group and model groups (n=8). **P*<0.05, ***P*<0.01.


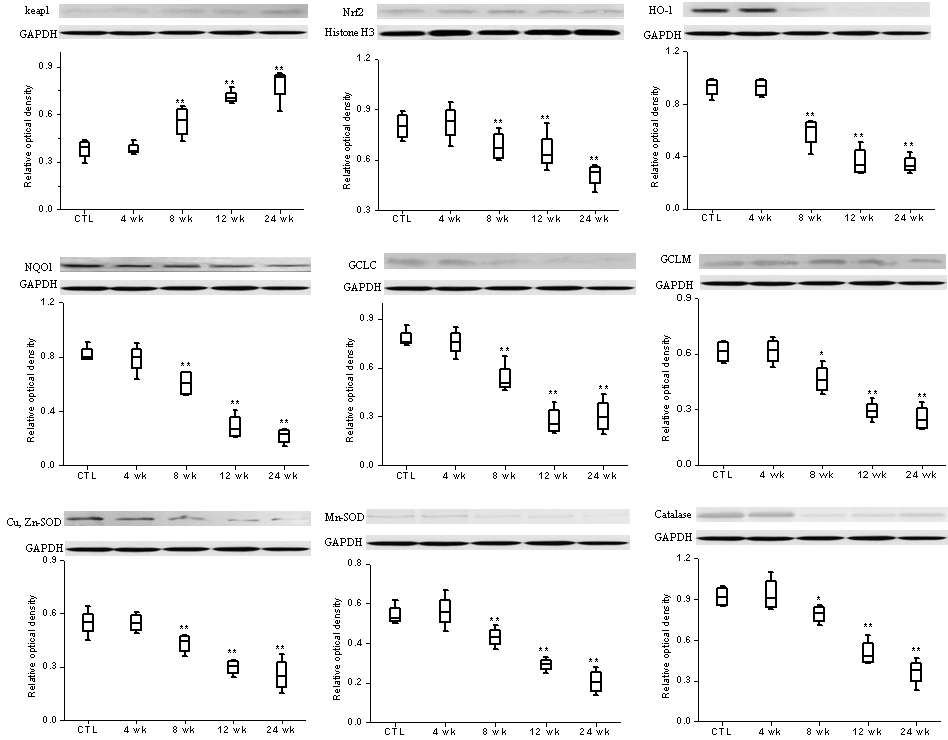


Figure S2 Anti-oxidative stress Nrf2 and its repressor keap1 and Nrf2 target protein expression in kidney from control and model rats. Representative western blots of anti-oxidative stress proteins including keap1, Nrf2, HO-1, NQO1, GCLC, GCLM, Cu,Zn-SOD, Mn-SOD and catalase in the control and model rats (n=8). **P*<0.05, ***P*<0.01.


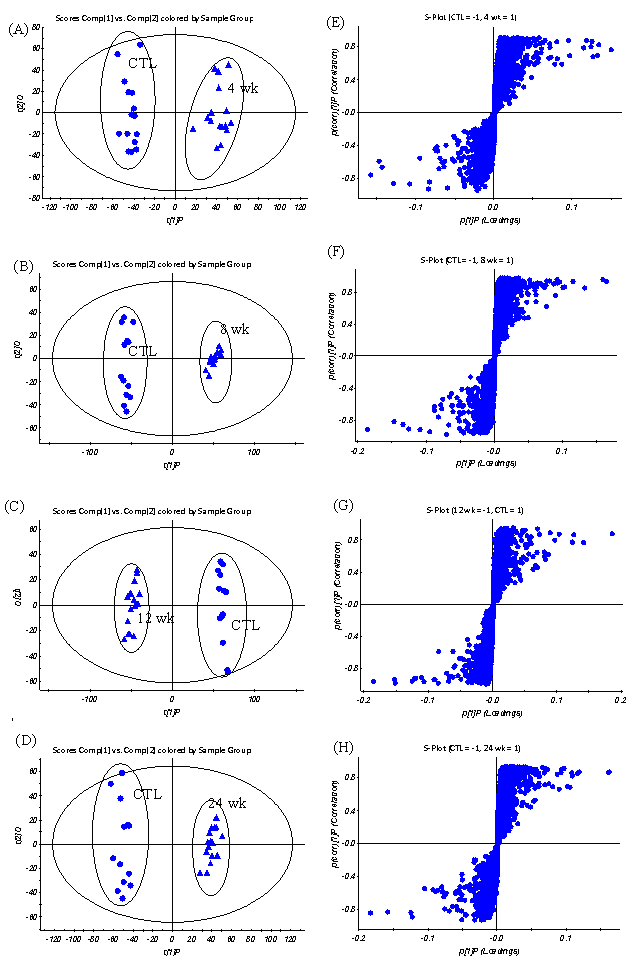


Figure S3 Results of multivariate analysis. The OPLS-DA score plots derived from the UPLC-MS profiles of kidney tissue extract in the control group and in the AAN group in the 4th week (A), 8th week (B), 12th week (C) and 24th week (D). S-plot based on kidney tissue profiling of the AAN and control group in the 4th week (E), 8th week (F), 12th week (G) and 24th week (H). The variables far from the origin contributed significantly to differentiate the clustering of AAN group from that of control group and were considered as potential biomarkers.


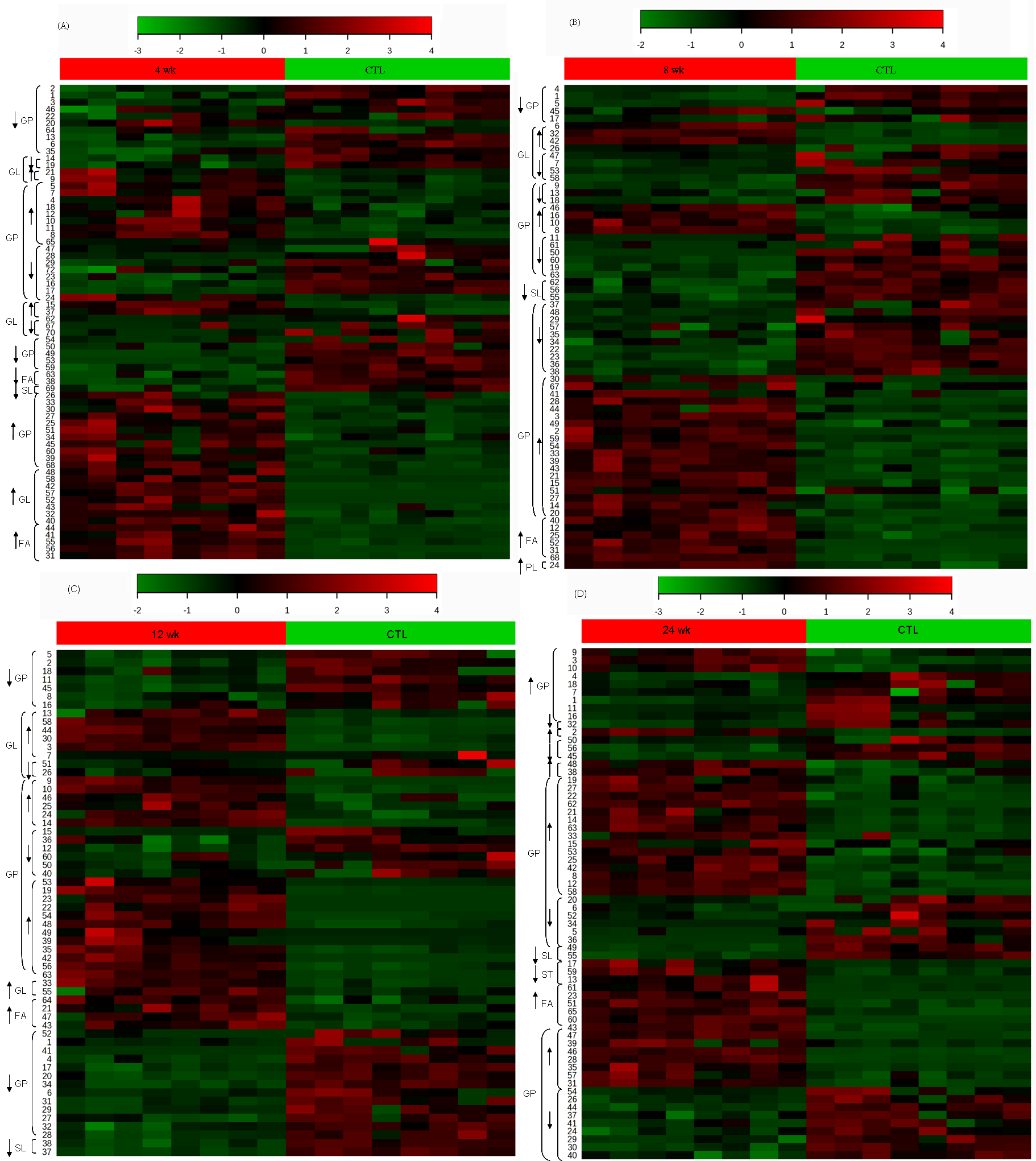


Figure S4 Heat maps for kidney tissue of the lipid metabolites. Heat maps for kidney tissue of the lipids in the 4th week (A), 8th week (B), 12th week (C) and 24th week (D). The color of each section is proportional to the significance of change of metabolites (red, upregulated; green, downregulated). Rows: lipid metabolites; Columns: kidney tissue samples.


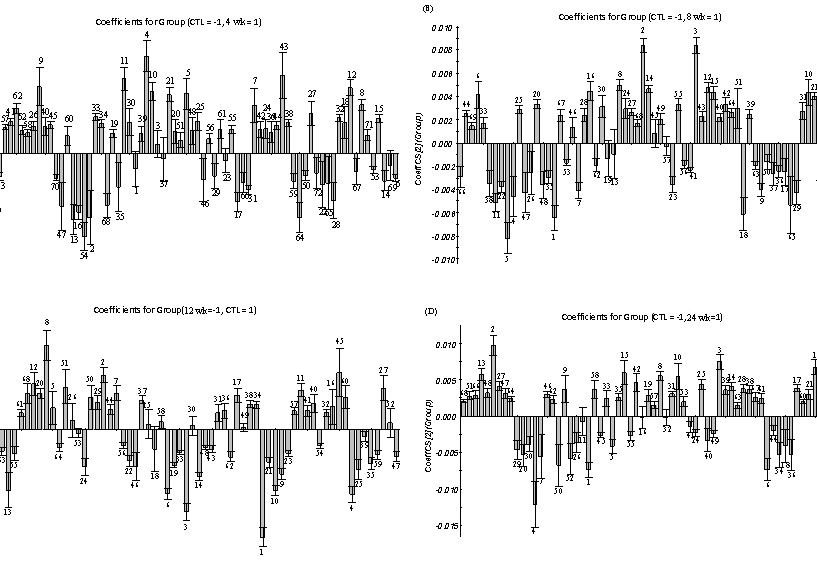


Figure S5 OPLS-DA loading plot from AAN group and control group in the 4th week (A), 8th week (B), 12th week (C) and 24th week (D). The loading plots represent which identified lipids were quantitatively higher or lower in AAN group compared with control group.


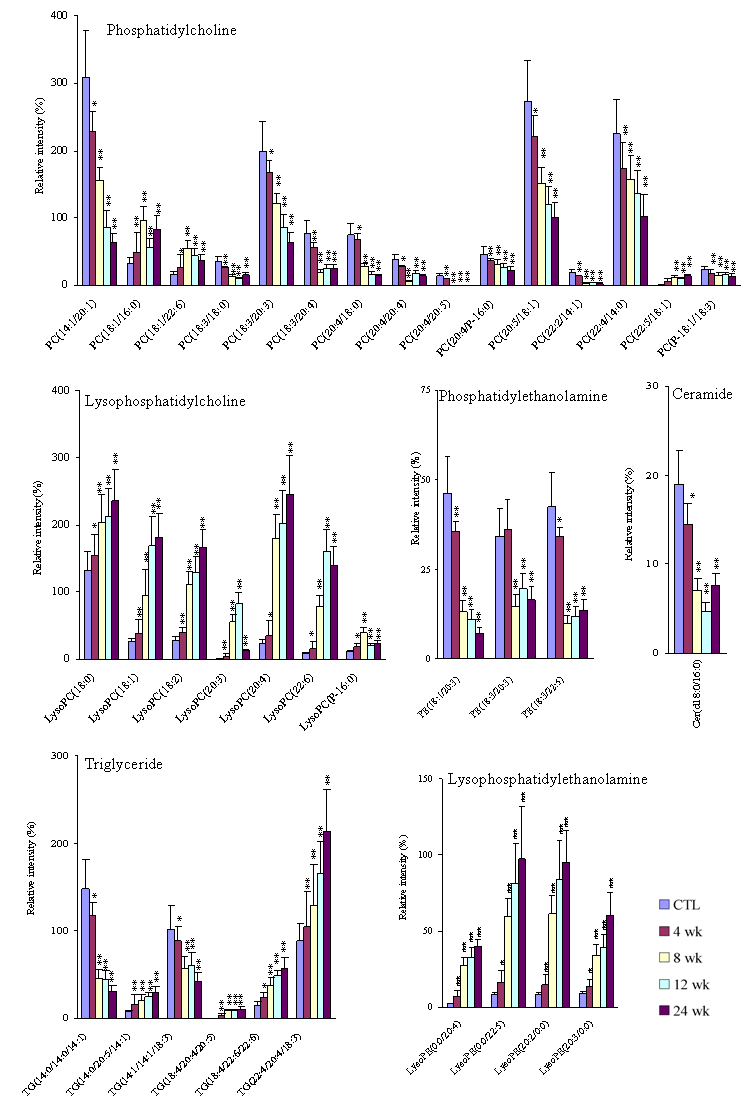


Figure S6 Quantification of individual lipid molecular species of glycerophospholipids, glycerolipids and sphingolipids of control group and AAN group in the 4th week, 8th week, 12th week and 24th week. Abundance is represented as the relative intensity (y axis) of an identified lipid (x axis). Significantly increased LysoPC(18:0), LysoPC(18:1), LysoPC(18:2), LysoPC(20:4), LysoPE(0:0/20:4), LysoPE(0:0/22:5), LysoPE(20:2/0:0),PE(18:1/20:3), TG(14:0/20:5/14:1), TG(18:4/20:4/20:5), TG(18:4/22:6/22:6), TG(22:4/20:4/18:3) as well as significantly decreased PC(14:1/20:1), PC(20:5/18:1), PC(18:3/20:3), PC(22:4/14:0), PC(20:4/P-16:0) and PC(20:4/18:0) were observed in the AAN rats.

Figure S7 A typical example workflow of metabolomics using UPLC-QTOF/MS as research tools for discovering and quantifying metabolites in complex mixtures.
